# Shared decision making in Australian physiotherapy practice: A survey of knowledge, attitudes, and self-reported use

**DOI:** 10.1371/journal.pone.0251347

**Published:** 2021-05-20

**Authors:** Tammy Hoffmann, Elizabeth Gibson, Christopher Barnett, Christopher Maher

**Affiliations:** 1 Institute for Evidence-Based Healthcare, Bond University, Gold Coast, Queensland, Australia; 2 John Hunter Outpatients, John Hunter Hospital, New Lambton Heights, New South Wales, Australia; 3 Department of Physiotherapy, University of Newcastle, Newcastle, New South Wales, Australia; 4 Institute for Musculoskeletal Health, The University of Sydney, Sydney, New South Wales, Australia; 5 Sydney Local Health District, Sydney, Australia; Endeavour College of Natural Health, AUSTRALIA

## Abstract

**Objective:**

To assess Australian physiotherapists’ knowledge about, attitudes towards, and self-reported use of shared decision making, as well as perceived barriers to its implementation in practice.

**Methods:**

Physiotherapists registered for a national Australian physiotherapy conference were invited via email and the conference app to complete a self-administered online questionnaire about shared decision making, including: a) knowledge, b) attitude to and reported approach in practice, c) behaviours used, d) barriers, e) previous training and future training interest. Responses were analysed descriptively and open-ended questions synthesised narratively.

**Results:**

372 physiotherapists (71% female, mean age 45 years, mean experience 23 years) completed the survey. Respondents had a good level of knowledge on most questions, with correct responses ranging from 39.5% to 98.5% of participants, and a generally positive attitude towards shared decision making, believing it useful to most practice areas. Sixty percent indicated they make decisions with their patients and there was general agreement between how decisions should be made and how they are actually made. The behaviour with the lowest reported occurrence was explaining the relevant research evidence about the benefits and harms of the options. The main perceived barriers were patient knowledge and confidence, consequent fewer physiotherapy sessions, and time constraints. Most (79%) were keen to learn more about shared decision making.

**Conclusions:**

Shared decision making is of growing importance to all health professions and rarely studied in physical therapy. This sample of Australian physiotherapists had a generally positive attitude to shared decision making and learning more about it. Opportunities for providing such skills training at the undergraduate level and in continuing professional development should be explored. This training should ensure that the communicating evidence component of shared decision making is addressed as well as debunking myths about perceived barriers to its implementation.

## Introduction

Shared decision making is a collaborative process, where the clinician and patient jointly participate in making a health decision after discussing the options, the pros and cons of these options, and taking into account the patient’s values, preferences, and circumstances [1]. It is also a useful process for incorporating discussions about research evidence into the discussion between clinician and patient [1].

There is an increasing expectation in Australia of greater collaborative decision making in health decisions between clinicians and patients, with national and state health bodies and agencies mandating such collaboration [2]. Shared decision making is one established process for such collaboration [2]. Much of the global research into shared decision making use has involved medical practitioners and their patients. However, there is increasing interest in, and expectation of, shared decision making being used by other health professionals [2], including physiotherapists [3–5].

The only known survey of physiotherapists about shared decision making was conducted in Germany [5] with 357 practising physiotherapists who completed an online survey examining knowledge, attitudes, and self-reported use of shared decision making and barriers to its implementation. Participants had limited knowledge about shared decision making, yet there was overall support for its use in physiotherapy and while most reported a positive attitude towards it, many reported using a paternalistic decision-making approach.

The aims of this study were to assess Australian physiotherapists’ knowledge about, attitudes towards, and self-reported use of shared decision making, as well as perceived barriers to its implementation in practice.

## Methods

### Participants

Eligible participants were physiotherapists registered to attend the Australian Physiotherapy Association (APA) biennial conference held in October 2017 in Sydney, Australia.

### Procedure

In the week prior to the conference, all registered delegates were sent an invitation by email, from APA staff, and via a notification in the conference app. The email/notification contained a research information statement explaining the purpose of the study and a link to an online survey (hosted on Survey Monkey platform). Consent was assumed if a participant responded to the survey. Ethics approval was provided by the Bond University Ethics Committee.

#### Survey

A survey was developed based on questions used in previous surveys of health professionals’ attitudes to shared decision making, including a study of German physiotherapists [5], medical students [6], medical residents [7] and medical specialists [8]. See S1 Appendix for a copy of the survey questions and response options. The survey was piloted with six physiotherapy colleagues who were not attending the conference. Some items were edited or added after the pilot, as indicated in the survey, including improvements for suitability to the Australian context. The survey consisted of five sections (no questions were compulsory):

1. Knowledge: Participants were asked to self-rate how much they know about shared decision making (11 point Likert scale, where 0 = I don’t know anything about it and 10 = comprehensive knowledge) [5] and 12 knowledge questions (true/false) [6].
2. Attitude: We used the modification used in the German physiotherapy study [5] of the Control Preference Scale [9] to measure attitude and reported approaches to decision making and listed five approaches classified into three styles [paternalistic (i.e. clinician-led decision-making), shared (i.e. shared decision making), informed (i.e. patient-led decision-making)]. Participants were also asked about the perceived usefulness (using a 5-point Likert scale from strongly disagree to strongly agree) of shared decision making for various patient groups [5].
3. Behaviours (for participants who currently worked clinically): Participants were asked who usually makes the treatment decision in a typical situation in their practice (from five options, that matched those used in an earlier question to measure attitude). Participants were also asked to reflect on the last consultation with a patient in which a treatment decision was made and indicate their agreement about whether they performed 11 behaviours that are part of the shared decision making process. Nine of these behaviours come from the Shared Decision-making Questionnaire (SDM-Q) [10] and we added two steps based on the shared decision making literature and piloting of the survey. We also included a single question about approach to shared decision making [6] that provided a clinical scenario (for this study, we chose the scenario of a patient with symptoms of lateral epicondylalgia) followed by a choice of four styles of decision-making.
4. Potential barriers to shared decision making: Participants were asked to rate a list of potential barriers (identified in the German study [5]) supplemented with additional possible barriers from the medical student survey [6] and the broader shared decision making literature. In addition to this list, in an open-ended question, participants were asked what they thought were the biggest barriers to using shared decision making in their practice.
5. Demographic questions: these included age, gender, highest level of physiotherapy qualification, years of physiotherapy experience, clinical area of practice, workplace setting, previous training in shared decision making (yes/no; if so, from where and if training in theory and/or practical skills with multiple responses allowed), and interest in learning more about it (5-point Likert scale from strongly disagree to strongly agree) [5,6].

Participants were also asked to provide any other comments they would like to make about shared decision making in physiotherapy, the results of which are not reported as there were no responses which had not already been captured elsewhere.

### Data analysis

Data are reported descriptively. Responses to the open-ended questions were synthesised narratively.

## Results

The invitation email was sent to 1720 conference registrants. While 372 participants commenced the survey (21% response rate), not every participant completed every question. For example, some questions about the use of shared decision making in clinical practice were only to be answered by physiotherapists who had worked clinically within the last two years (77%, n = 288). Question completion rates ranged from 70% -100%. Participants’ characteristics are presented in Table 1.

**Table 1:** Participant characteristics.

Characteristic	n (%)*
*Female*	200/283 (70.7)
*Mean age* (SD, range)	45.0 (11.2, 22–80)
*Years worked as physiotherapist Mean (SD)*	23.1 (22.7)
*Highest physiotherapy qualification*	n = 283
• Bachelor degree	106 (37.5)
• Masters degree	87 (30.7)
• PhD	50 (17.7)
• Other (e.g. postgraduate diploma, postgraduate certificates)	44 (15.6)
• Graduate entry masters degree	11 (3.9)
• Doctoral (coursework) degree	7 (2.5)
• Current physiotherapy student	2 (0.7)
*Main area of practice*	n = 280
• Chronic musculoskeletal conditions	140 (50.0)
• Acute musculoskeletal conditions	116 (41.4)
• Orthopaedic conditions	90 (32.1)
• Sports physiotherapy	76 (27.1)
• Manipulative physiotherapy	49 (17.5)
• Other (please specify)	47 (16.8)
• Neurological conditions	46 (16.4)
• Gerontology	42 (15.0)
• Paediatrics	32 (11.4)
• Cardiopulmonary conditions	29 (10.4)
• Women’s health (including obstetrics and gynaecology)	25 (8.9)
• General (work across all areas)	24 (8.6)
• Ergonomics and occupational health	16 (5.7)
• Health promotion	12 (4.3)
*Current work setting*	n = 281
• Private practice	126 (44.8)
• University—teaching and/or research	67 (23.8)
• Hospital—mainly outpatient caseload	55 (19.6)
• Hospital—mainly inpatient caseload—acute care	40 (14.2)
• Other (please describe)	40 (14.2)
• Community care	24 (8.5)
• Hospital—mainly inpatient caseload—rehabilitation	20 (7.1)
• Sport organisation/setting	11 (3.9)
• School/educational organisation	3 (1.1)
*Participants not practising in Australia*	n = 282 16 (5.7)

* number of respondents to each question varied.

### Knowledge about shared decision making

Sixty-nine (19%) participants indicated that they had no knowledge of the concept of shared decision making and nine participants (2.4%) reported comprehensive knowledge. The mean response was 5.9 (SD 2.7), on an 11-point scale. Table 2 shows the results for true or false responses to statements about shared decision making (n = 327, 88%). Nearly all participants (99%) correctly answered two questions [to promote shared decision making, a clinician will 1) support the patient in becoming informed and comparing options and 2) should indicate that alternative treatment or management options exist (including that one option may be ‘no action‘)]. The question with the lowest level of correct responses (40%) was that shared decision making increases the length of a consultation.

**Table 2:** Knowledge about shared decision making.

Question (whether answer is true/false)	% answering correctly (ranked highest to lowest)
To promote shared decision making, a clinician will support the patient in becoming informed and comparing options (true)	98.5
To promote shared decision making, a physiotherapist should indicate that alternative treatment or management options exist (including that one option may be ‘no action‘) (true)	98.5
Whenever possible, a physiotherapist should integrate the patient’s preferences when deciding what to do next (true)	98.2
By doing shared decision making, patients may be more likely to adhere to the chosen treatment plan (true)	96.0
Access to decision support tools that summarise the evidence-based benefits and harms of treatment options for different conditions/problems would be helpful (true)	93.6
Whenever possible, I should try to explain the natural history of a condition to patients and what might happen without active treatment (true)	93.3
Shared decision making causes patients to feel uncertain about their decisions (false)	88.7
Understanding the mechanism or pathophysiology of how a treatment works is more important than having evidence about the treatment’s effect (false)	72.2
There is not enough evidence about the effectiveness of some physiotherapy treatments. This makes talking with patients about treatment options and the advantages and disadvantages of the options difficult (true)	56.9
Most people will understand natural frequency (e.g., 1 in every 100 people) better than a percentage (true)	53.8
When communicating information about risks, it is best to use relative risk (false)	47.1
Doing shared decision making will increase the length of a visit/consultation (false)	39.5

### Attitude towards shared decision making

When asked their opinion about how healthcare decisions should be made, just over half of participants (57%, n = 211) chose the option that the patient and therapist should share responsibility for the making the final treatment decision together and 28% (n = 103) indicated that the patient should make the final decision after considering the therapist’s opinion. A small number indicated that the patient should make the final decision (8%), that the therapist should make the final decision after considering the patient’s opinion (7%), or that the therapist should make the final decision (0.3%).

**Fig 1:**
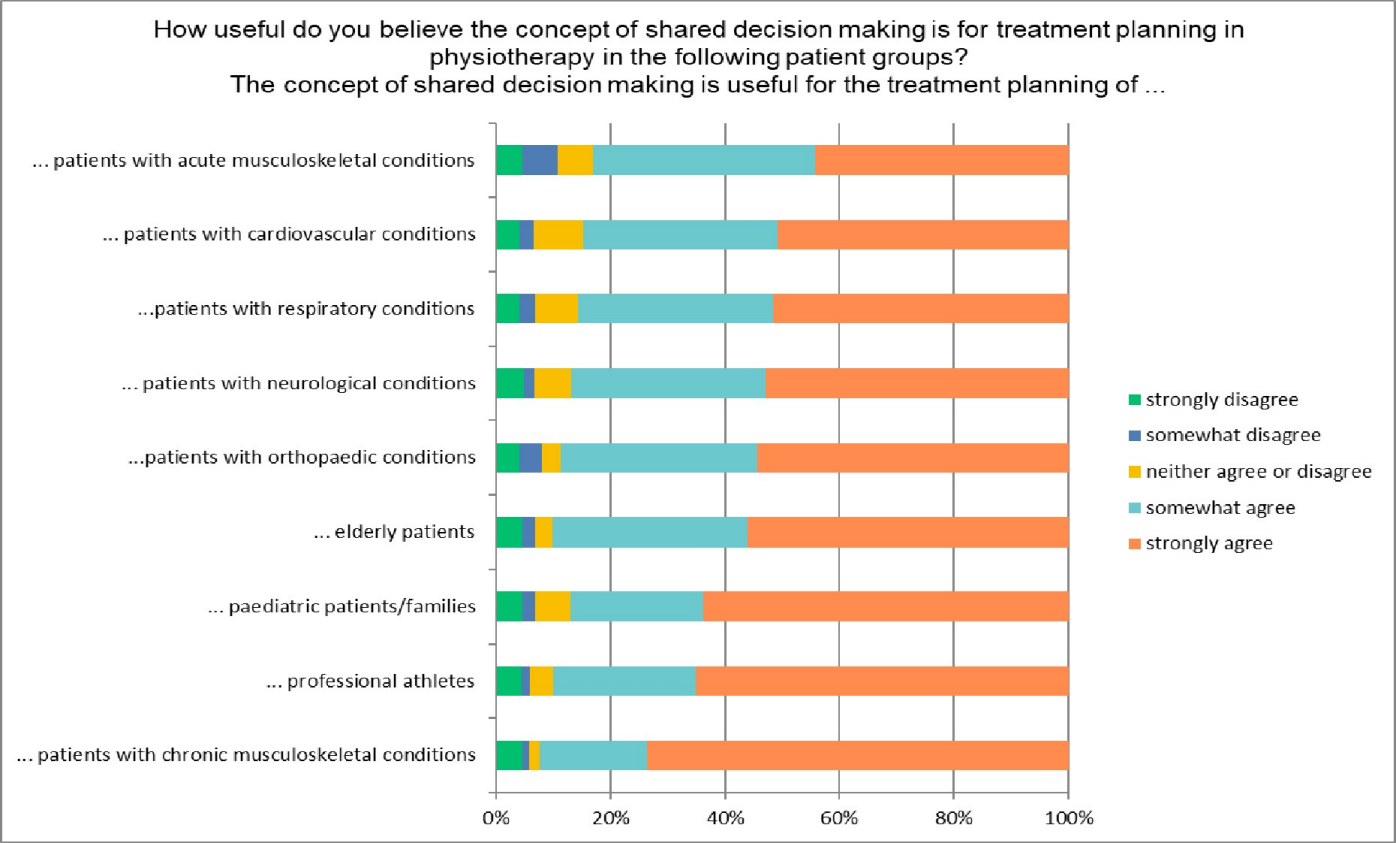
Percentage of participants agreeing or disagreeing with the perceived usefulness of shared decision making for various groups of patients.

Fig 1 shows participants’ responses to the perceived usefulness of shared decision making for treatment decisions for various patient groups. The general agreement was similar across the groups, although there was some variation in the extent of agreement. For example, 73% strongly agreed that it was useful for patients with chronic musculoskeletal conditions, whereas 44% indicated strong agreement for patients with acute musculoskeletal conditions.

### Shared decision making behaviours

Participants who worked clinically (n = 294) were asked who usually makes the treatment decision in a typical situation in their practice. Over half of participants (60%; n = 175) indicated they make the decision together with their patient; 24% (n = 71) indicated they make the decision on their own after considering the patient’s opinion; 13% (n = 38) reported that the patient makes the treatment decision after seriously considering the physiotherapists’ opinion. The remaining options at the extremes of continuum (“I make the decision on my own” and “the patient makes the decision on his/her own”) were each chosen by 1.7% (n = 5) participants.

In response to the clinical scenario, most (86%, n = 244) indicated they would use an approach that was most reflective of shared decision making (i.e. ‘I would share evidence-based information with the patient, and elicit his/her preference so that we make an informed decision together’), followed by 8.8% (n = 25) who ‘would share evidence-based information with the patient, and allow him/her to make the decision on their own’.

**Fig 2:**
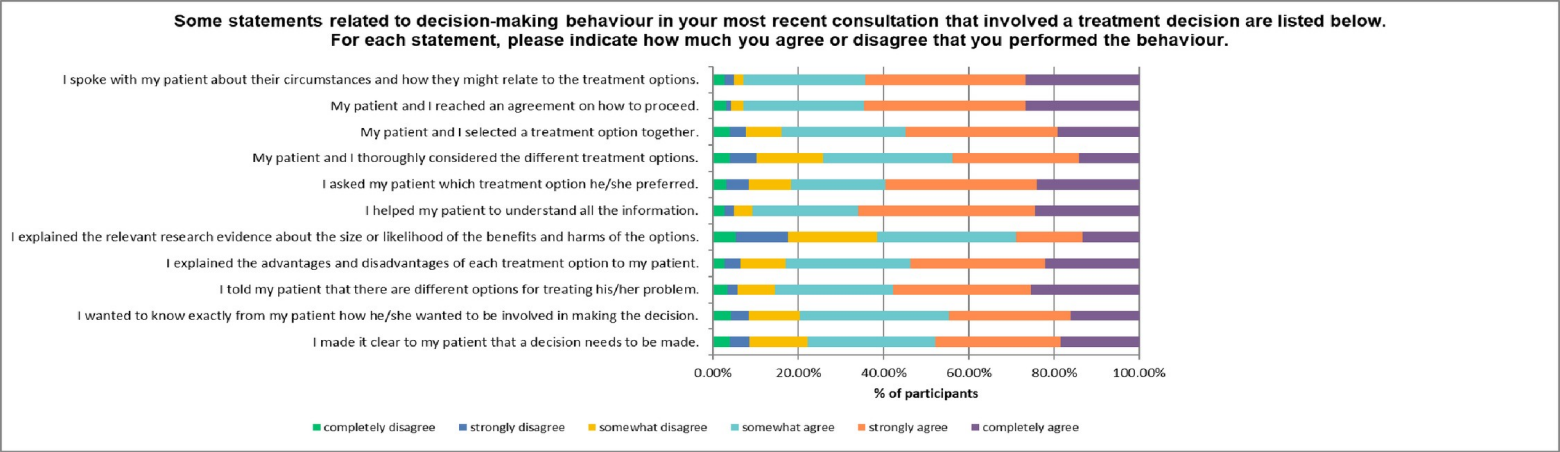
Percentage of participants agreeing or disagreeing with whether they performed various shared decision making behaviours during their most recent consultation.

Participants were asked to reflect on their most recent consultation that involved a treatment decision and indicate how much they agreed they undertook each of the listed behaviours (Fig 2). The most frequently reported behaviours were reaching an agreement with the patient on how to proceed and speaking with the patient about their circumstances and how these related to treatment options. The behaviour with the lowest reported occurrence was explaining the relevant research evidence about the size or likelihood of the benefits and harms of the options.

### Perceived barriers to implementing shared decision making

**Fig 3:**
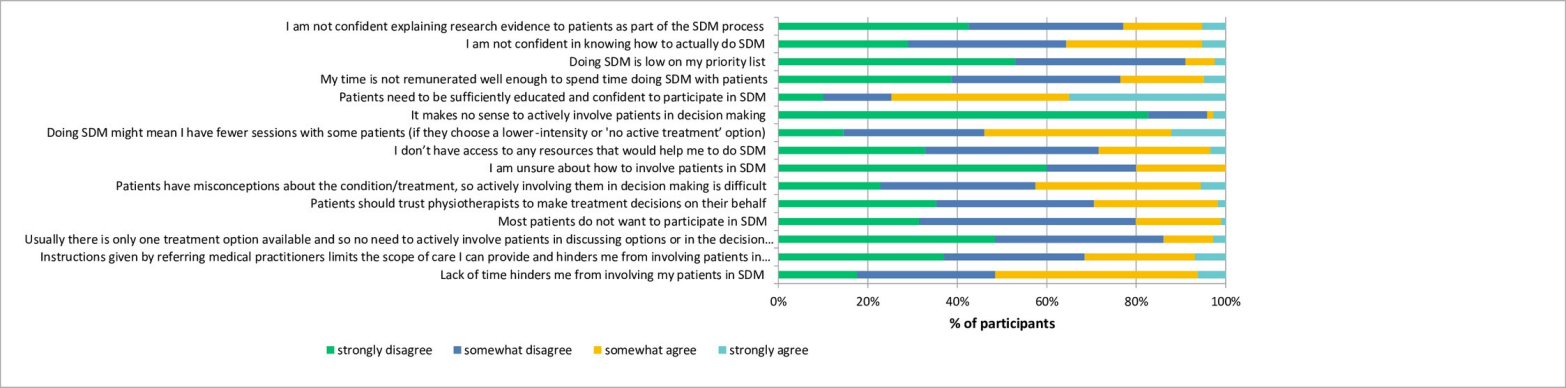
Perceived barriers to shared decision making—percentage of participants agreeing or disagreeing with statements.

Fig 3 shows participants’ agreement with potential barriers. There was highest agreement (strongly or somewhat agree) with the suggested barrier that patients need to be sufficiently educated and confident to participate in shared decision making (76%), that doing shared decision making may mean fewer physiotherapy sessions (56%), and that lack of time was a barrier to doing shared decision making (52%). Most participants disagreed (strongly or somewhat disagree) with the potential barriers that it makes no sense to involve patients in decision-making (96%), that shared decision making was a low priority for them (91%), and that there is usually only one option available and therefore no need to actively involve patients (86%). Table 3 summarises the barriers, along with some illustrative quotes, suggested by participants in response to the open-ended question, with time and patient expectations the most frequently nominated barriers.

**Table 3:** Biggest perceived barriers to shared decision making.

Barriers	Illustrative quotes
Time (within the consultation)	“…*this would involve me educating patients a lot about their condition*, *clearing past misconceptions to their condition*, *… and physiotherapy management*.”
Time and timeliness of decisions (particularly for acute settings)	*“…if the situation is acute and a decision needs to be made within a short time frame*. *Patients or parents may be too overwhelmed to be part of the decision process*. *Some parents are unable to understand information provided because they are under a lot of stress*.* *.* *.*” and have a lot of things on their mind”*. *“with acute patients- time limits discussing the full scope of options for patients… with chronic patients…I have longer session times”*.
Time (to learn how to do shared decision making; to search for and read evidence)	*“…time to go keep up with research evidence on different PT modalities and treatment options (rather than the time spent with the patient)*.*”*
Patient expectations, attitudes, beliefs, and misconceptions	*“expectations of patients to spend less time talking so treatment can be done”* *“they expect you are the expert and will choose the correct treatment”* *“preconceived patient ideas or beliefs”* *“patient misconceptions reinforced by misinformation and…practitioner is left with the dilemma of either providing sub optimal treatment at patient request or providing optimal treatment against the wishes of the patient*.*”*
Insufficient health literacy or cognitive capacity of some patients to engage in shared decision making	*“patient’s cognitive reasoning ability… especially in acute wards in hospitals*, *with the elderly who can be easily confused*, *or those who have decision overload due to various health/life circumstances”*
Other system or organisational barriers	Team-based decisions—e.g. *“you are not the only person involved in treatment*, *often need multidisciplinary team*.” Dependent on other providers—e.g. *“surgeon protocols (which) limit treatment options”* Work in models which restrict the care provided–e.g. *“I worked in Aged Care so what physios were allowed to do was often determined by ACFI [Aged Care Funding Instrument] and how much each facility was prepared to spend on physio”*.
Lack of modelling of shared decision making in practice and awareness of what it consists of	*“this not being well modelled in practice and so it is not routine to go by each step”* “*I think I understand the concept yet it now seems that SDM is a constructed tool & training is required–it’s moved beyond informed choice”* *“… I am probably not using the method to the extent that is suggested in this survey”*

### Training in shared decision making

Of the 261 (70%) participants who identified previously learning about shared decision making, the most common sources were reading about it (53%), at a training session or conference (40%), from colleagues (33%), and from university studies (22%). When asked about any previous training, 24% of respondents reported they had receiving training in the theory of it and only 16% reported receiving practical/skill training. Many participants (79%, n = 286) agreed or strongly agreed that they wanted to learn more about how to do shared decision making with patients.

## Discussion

This survey of Australian physiotherapists about shared decision making found a moderate level of self-reported knowledge and good level of knowledge for most questions, an indication by about half of the participants that it was the preferred approach to decision-making, agreement by the majority that it was useful across multiple areas of physiotherapy practice, and self-report by about half of participants that they had performed the majority of shared decision making behaviours that are identified as best practice.

There was general agreement between participants’ responses to how health decisions should be made and how they actually are made, with about half indicating that responsibility for the final treatment decision should be shared between patient and therapist. In the survey of German physiotherapists [5], there was a difference between attitudes toward and reported use of decision-making approaches. While about half reported a preference for a shared decision making approach, two-thirds reported typically using a paternalistic style and 29% reported doing shared decision making. Mean reported knowledge (5.9) in our sample was also higher than in the study of German physiotherapists (mean 1.3) [5], which may be partly explained by greater awareness and reported use of shared decision making amongst Australian physiotherapists.

When reflecting on a recent consultation, most participants at least somewhat agreed that they had performed most of the shared decision making behaviours listed. The behaviours with the lowest reported occurrence were explaining the relevant research evidence about the size or likelihood of the benefits and harms of the options and considering all of the options. The two knowledge questions that corresponded to the behaviour of communicating evidence were among the questions with the lowest percentage of correct responses, with approximately half answering incorrectly, yet only about one quarter indicated lack of confidence in these skills.

Although many shared decision making behaviours were reported as occurring, this was assessed using self-report questions and may not reflect actual behaviour which can only be accurately captured by observing consultations. In two studies of physiotherapists that have done this, low levels of shared decision making (out of a possible 100, mean OPTION score of 24 [11] and 5.6 [12]) were observed.

Many of the reported barriers to shared decision making, such as time, patient expectations, patient capacity, and system barriers are similar to those that have been reported in other studies, both in physiotherapy [5] and other disciplines [13–15]. A concern of many participants was that involving patients in shared decision making might mean fewer sessions if patients choose a lower intensity or no active treatment option. This concern may reflect the fee for service payment arrangements that apply to many Australian physiotherapists and is not a barrier that been explicitly explored in other studies. Previously identified financial barriers to shared decision making in studies of medical professionals have been inadequate reimbursement for the time needed for shared decision making [13], and for conducting a collaborative discussion about whether a procedure is needed, compared to the fee for performing a procedure [16]. As shared decision making can result in reduced uptake of options that do not have clear benefits for all [17], funding systems based on fee-for-service models can conflict with the goals and outcomes of shared decision making and research is needed on these possible unintended effects of its implementation [18]. The highest ranked barrier in the survey of German physiotherapists was constraints imposed by the referring physician’s instructions, ranked lower (sixth) in our study and likely reflects the greater level of autonomy that Australian physiotherapists typically have.

Some of the highest-ranked barriers reflect common myths about shared decision making, including the belief that patients need to be sufficiently educated to participate in shared decision making, which was also found in a recent survey of medical students [19]. Patients’ educational background or ability is not such an insurmountable barrier as the respondents may have presumed. It has been shown that both patients and clinicians can successfully learn the set of behaviours needed for collaborative decision-making [1]. It is important that health inequalities are not exacerbated by not attempting to involve disadvantaged patients in decisions about their healthcare [1]. The misunderstanding that shared decision making makes consultations impracticably longer was prevalent. However systematic reviews report either no [16], uncertain [17] or minimal (median increase of 2.6 minutes) [20] effect on consultation length. Some responses to the open-ended questions, as well as the discrepancy between confidence in certain behaviours and responses to knowledge questions, indicate that some participants believe they are performing shared decision making, when they may not be. This misunderstanding about what it is and what is involved has been noted elsewhere [1,21], with clinicians often assuming it is mostly about good communication and patient education. While these are related, shared decision making brings together patient-centred communication and evidence-based practice [1], and involves explicitly listing all the options, discussion about and quantification of the benefits and harms of each option, and an invitation to participate in the decision-making process.

We are aware of only one other survey of Australian health professionals about shared decision making [22]. It surveyed a convenience sample of attendees of a shared decision making masterclass about perceived barriers to implementation. Respondents included clinicians of various disciplines, consumers, managers and policy officers. Barriers identified were similar to those in our survey, namely time constraint, lack of resources and low knowledge, confidence and skills. The need for more modelling of shared decision making in practice, especially given that some clinicians are confident they are already doing shared decision making while misunderstanding what is actually involved, was also noted.

### Limitations

In addition to the self-report of behaviours, other study limitations are the low response rate and that participants may not be representative of all physiotherapists as those with an interest in shared decision making could have been more likely to complete the survey and those who attend conferences may be more aware of evidence-based issues and shared decision making. Our sample had a higher proportion with a higher research degree (18% vs 2% of APA members) (Australian Physiotherapy Association of Australia, 2020, unpublished membership demographic data), and on average, about 10 more years’ experience than the general physiotherapy population [23], but was comparable to Australian physiotherapists on most other demographics. In our survey, most questions focussed on shared decision making for treatment decisions, whereas it is also appropriate for test and screening decision-making. Another limitation is that knowledge was assessed with closed-ended questions (such as true or false response options), rather than more detailed or nuanced methods of knowledge assessment.

In this sample of Australian physiotherapists, there was a generally positive attitude to shared decision making and enthusiasm for learning more about how to do it. However, only about half indicated that responsibility for the final treatment decision should be shared between patient and therapist. A critical step in shared decision making, explanation of the benefits and harms of treatments, was reportedly lacking. Perceived barriers to implementing shared decision making were generally unfounded. Opportunities for providing skills training at the undergraduate level and in continuing professional development should be explored. Key aspects that should be addressed by this training include: how to communicate evidence, clarifying what shared decision making is and is not, role modelling of it, as well as debunking myths about perceived barriers to its implementation.

## Supporting information

S1 Appendix Shared decision making in physiotherapy survey. (PDF)
