# Supplementary material for: Shared decision making in Australian physiotherapy practice: A survey of knowledge, attitudes, and self-reported use
Source: PLoS One. 2021 May 20;16(5):e0251347. doi: 10.1371/journal.pone.0251347 (PMC8136718; doi:10.1371/journal.pone.0251347)
Supplement: S1 Appendix — (PDF) [file pone.0251347.s001.pdf]

Shared Decision-Making in Physiotherapy Survey

1. How much do you know about the concept of shared decision making? *

I don't know anything about the concept. … I have comprehensive knowledge on the concept.

❑	❑	❑	❑	❑	❑	❑	❑	❑	❑	❑
0	1	2	3	4	5	6	7	8	9	10

2. If you have learnt about shared decision making, where did this occur? * (Multiple responses allowed)

- ❑ During a training session or a conference.
- ❑ During physiotherapy university education.
- ❑ I read about it.
- ❑ Colleagues have told me about it.
- ❑ Other (please specify)

3. Have you received theoretical training in shared decision making (e.g. a lecture)? †

- ❑ Yes
- ❑ No

4. Have you received practical training in shared decision making (e.g. using role plays, simulated patients, etc) †

- ❑ Yes
- ❑ No

5. In your opinion, how should healthcare decisions be made? ‡

- ❑ As the physiotherapist, I should make the final decision about which treatment the patient should receive.
- ❑ As the physiotherapist, I should make the final decision about which treatment the patient should receive after seriously considering the patient's opinion.
- ❑ The patient and I should share responsibility for making the final treatment decision together.
- ❑ The patient should make the final decision about which treatment he/she would receive after seriously considering my opinion.
- ❑ The patient should make the final decision about which treatment he/she would receive.

6. How useful do you believe the concept of shared decision making is for treatment planning in physiotherapy in the following patient groups? The concept of shared decision making is useful for the treatment planning of ... §

	strongly disagree	somewhat disagree	neither agree or disagree	somewhat agree	strongly agree
… patients with *acute* musculoskeletal conditions	❑	❑	❑	❑	❑
… patients with *chronic* musculoskeletal conditions.	❑	❑	❑	❑	❑
… ... paediatric patients/families§	❑	❑	❑	❑	❑
… patients with cardiovascular conditions	❑	❑	❑	❑	❑
… patients with respiratory conditions§	❑	❑	❑	❑	❑
… patients with neurological conditions	❑	❑	❑	❑	❑
… patients with orthopaedic conditions§	❑	❑	❑	❑	❑
… elderly patients	❑	❑	❑	❑	❑
… professional athletes	❑	❑	❑	❑	❑
… others. (Please specify) ____________________	❑	❑	❑	❑	❑

7. Who usually makes the treatment decision in clinical practice? Please consider a typical situation in your clinical practice when answering the question. ‡

Please consider a typical situation in clinical practice when answering the question.

❑ I make the treatment decision on my own.
❑ I make the treatment decision on my own after considering my patient’s opinion.
❑ I make the treatment decision together with my patient.
❑ The patient makes the treatment decision after seriously considering my opinion.
❑ The patient makes the treatment decision on her/his own.
❑ Not applicable – I do not work clinically.

8. These questions relate to shared decision making. Please indicate whether you feel each of the following statements is TRUE or FALSE. **

Shared decision making causes patients to feel uncertain about their decisions.	☐ True ☐ False
Doing shared decision making will increase the length of a visit/consultation.**	☐ True ☐ False
Most patients will understand natural frequency (e.g., 1 in every 100 people) better than a percentage (e.g 10% of people).	☐ True ☐ False
Understanding the mechanism or pathophysiology of how a treatment works is more important than having evidence about the treatment's effect.**	☐ True ☐ False
There is not enough evidence about the effectiveness of some physiotherapy treatments. This makes talking with patients about treatment options and the advantages and disadvantages of the options difficult.**	☐ True ☐ False
When communicating information about risks, it is best to use relative risk (e.g. double the risk of an adverse event occurring from a treatment).	☐ True ☐ False
To promote shared decision making, a clinician will support the patient in becoming informed and comparing options.	☐ True ☐ False
Whenever possible, a physiotherapist should integrate the patient's preferences when deciding what to do next.	☐ True ☐ False
To promote shared decision making, a physiotherapist should indicate that alternative treatment or management options exist (including that one option may be 'no action').	☐ True ☐ False
By doing shared decision making, patients will be more likely to adhere to the chosen treatment plan.**	☐ True ☐ False
Whenever possible, I should try to explain the natural history of a condition to patients and what might happen without active treatment.**	☐ True ☐ False
Access to decision support tools that summarise the evidence-based benefits and harms of treatment options for different conditions/problems would be helpful.	☐ True ☐ False

9. Have you worked clinically within the last 2 years?

- ❑ Yes
- ❑ No

If answered yes to question 9, proceed to question 10

10. Please think of the last treatment session in which a treatment decision was made.

What health complaint/problem/illness was the treatment session about?

What treatment was decided upon? *

11. Some statements related to the decision making in the above consultation are listed below. For each statement, please indicate how much you agree or disagree. ††

	completely disagree	strongly disagree	somewhat disagree	somewhat agree	strongly agree	completely agree
I made it clear to my patient that a decision needs to be made.	❑	❑	❑	❑	❑	❑
I wanted to know exactly from my patient how he/she wanted to be involved in making the decision.	❑	❑	❑	❑	❑	❑
I told my patient that there are different options for treating his/her problem.	❑	❑	❑	❑	❑	❑
I explained the advantages and disadvantages of each treatment option to my patient.	❑	❑	❑	❑	❑	❑
I explained the relevant research evidence about the size or likelihood of the benefits and harms of the options. ††	❑	❑	❑	❑	❑	❑
I helped my patient to understand all the information.	❑	❑	❑	❑	❑	❑
I asked my patient which treatment option he/she preferred.	❑	❑	❑	❑	❑	❑
My patient and I thoroughly weighed the different treatment options.	❑	❑	❑	❑	❑	❑
My patient and I selected a treatment option together.	❑	❑	❑	❑	❑	❑
My patient and I reached an agreement on how to proceed.	❑	❑	❑	❑	❑	❑
I spoke with my patient about their circumstances and how they might relate to the treatment options. ††	❑	❑	❑	❑	❑	❑

12. For each of the following statements, please indicate how much you agree or disagree. ‡‡

	strongly disagree	somewhat disagree	somewhat agree	strongly agree
Lack of time hinders me from involving my patients in SDM *	❑	❑	❑	❑
Instructions given by referring medical practitioners limits the scope of care I can provide and hinders me from involving patients in decision making *	❑	❑	❑	❑
Usually there is only one treatment option available and so no need to actively involve patients in discussing options or in the decision making *	❑	❑	❑	❑
Most patients do not want to participate in SDM *†	❑	❑	❑	❑
Patients should trust physiotherapists to make treatment decisions on their behalf †	❑	❑	❑	❑
Patients have misconceptions about the condition/treatment, so actively involving them in decision making is difficult *	❑	❑	❑	❑
I am unsure about how to involve patients in SDM ‡‡	❑	❑	❑	❑
I don’t have access to any resources that would help me to do SDM †	❑	❑	❑	❑
Doing SDM might mean I have fewer sessions with some patients (if they choose a lower-intensity or 'no active treatment’ option) ‡‡	❑	❑	❑	❑
It makes no sense to actively involve patients in decision-making *	❑	❑	❑	❑
Patients need to be sufficiently educated and confident to participate in SDM †	❑	❑	❑	❑
My time is not remunerated well enough to spend time doing SDM with patients ‡‡	❑	❑	❑	❑
Doing SDM is low on my priority list †	❑	❑	❑	❑
I am not confident in knowing how to actually do SDM ‡‡	❑	❑	❑	❑
I am not confident explaining research evidence to patients as part of the SDM process ‡‡	❑	❑	❑	❑

13. What do you think are the biggest barriers to using shared decision making in your practice?

[free text response]

14. Please read the following scenario and then indicate which decision style you would use if you were in this situation. There are no right or wrong answers. †

A 35 year old female presents to your practice with symptoms of tennis elbow/lateral epicondylalgia. She is unsure of whether she should undertake physiotherapy treatment, an oral non-steroidal anti-inflammatory drug she has read about online, or a steroid injection that her colleague had when they had tennis elbow. You conduct a clinical assessment.Imagine that you are the therapist in this situation, how would you respond? (choose one response)

- ❑ I would use evidence-based information to decide on the best course of action for the patient and inform the patient of my decision.
- ❑ I would share evidence-based information with the patient, and elicit his/her preference so that we make an informed decision together.
- ❑ I would share evidence-based information with the patient and allow him/her to make the decision on their own.
- ❑ I would share evidence-based information with the patient and choose the best course of action for him/her.

15. Please indicate how much you agree or disagree with the following statement. I would like to know more about how to do shared decision making with patients. †

Strongly disagree	Disagree	Uncertain	Agree	Strongly agree
❑	❑	❑	❑	❑

16. How old are you? (in years)

17. What is your gender?

❑ Female   ❑ Male

18. What is your highest physiotherapy qualification(s)?

❑ Bachelor degree
❑ Masters degree
❑ PhD
❑ Graduate entry masters degree
❑ Doctoral degree
❑ Current physiotherapy student
❑ Other (please specify)

19. In which state or territory do you currently live? (of Australia)
or country other than Australia? (options provided)

20. How many years have you worked as a physiotherapist?

21. On average, how many hours per week do you currently work as a physiotherapist?*

22. If you work clinically, on average, how long (in minutes) is a typical treatment session? *

23. What is your main area/s of practice? (Multiple responses allowed) *

❑ Chronic musculoskeletal conditions
❑ Acute musculoskeletal conditions
❑ Orthopaedic conditions
❑ Sports physiotherapy
❑ Manipulative physiotherapy
❑ Neurological conditions
❑ Gerontology
❑ Paediatrics
❑ Cardiopulmonary conditions
❑ Women's health (including obstetrics and gynecology)
❑ General (work across all areas)
❑ Ergonomics and occupational health
❑ Health promotion
❑ Other (please specify)

24. In which work setting do you currently work? (Multiple responses allowed) *

❑ Private practice
❑ University - teaching and/or research
❑ Hospital - mainly outpatient caseload
❑ Hospital - mainly inpatient caseload - acute care
❑ Community care
❑ Hospital - mainly inpatient caseload - rehabilitation
❑ Sport organisation/setting
❑ School/educational organisation
❑ Other (please describe)

25. Are there any other comments you would like to make about shared decision making in physiotherapy?

References

1. Degner LF, Sloan JA. Decision making during serious illness: What role do patients really want to play? Journal of Clinical Epidemiology. 1992;45:941-50.
2. Degner LF, Sloan JA, Venkatesh P. The Control Preferences Scale. Canadian Journal of Nursing Research. 1997;29(3):21-43.
3. Durand M-A, Yen R, Barr PJ, Cochran N, Aarts J, Légaré F, et al. Assessing medical student knowledge and attitudes about shared decision making across the curriculum: protocol for an international online survey and stakeholder analysis. BMJ Open. 2017;7(6):e015945-e. doi: 10.1136/bmjopen-2017-015945.
4. Scholl I, Kriston L, Dirmaier J, Buchholz A, Härter M. Development and psychometric properties of the Shared Decision Making Questionnaire - physician version (SDM-Q-Doc). Patient Education and Counseling. 2012;88(2):284-90. doi: 10.1016/j.pec.2012.03.005.
5. Topp J, Westenhöfer J, Scholl I, Hahlweg P. Shared decision-making in physical therapy: A cross-sectional study on physiotherapists' knowledge, attitudes and self-reported use. Patient Education and Counseling. 2018;101(2):346-51. doi: 10.1016/j.pec.2017.07.031.

Sources of questions when used in previous studies

* from or adapted from Topp et al. (2018) survey

[†] from or adapted from Durand et al. (2017) survey

[‡] adapted from versions of The Control Preferences Scale (CPS) (Degner et al. 1992; 1997) used in Topp et al. (2018) and Durand et al. (2017)

[§] adapted from Topp et al. (2018) survey with additional items (noted as [§]) based on pilot of survey and literature and for Australian context.

** 8 items from or adapted from Durand et al. (2017) survey with additional items as noted ([**]) based on pilot of survey and literature.

[††] 9 items from Topp et al. (2018) survey who used the SDM-Q (Scholl et al. 2012), except items 5 and 11 (noted as [††]) which we added based on literature and piloting of the survey.

[‡‡] a mixture of items from or adapted from Topp et al. (2018) (noted as *) and from Durand et al. (2017) (noted as †) surveys, with four additional items added based on literature and piloting (noted as [‡‡]).
